# Ambient air pollution and cardiovascular disease rate an ANN modeling: Yazd-Central of Iran

**DOI:** 10.1038/s41598-021-94925-8

**Published:** 2021-08-20

**Authors:** Mahrokh Jalili, Mohammad Hassan Ehrampoush, Mehdi Mokhtari, Ali Asghar Ebrahimi, Faezeh Mazidi, Fariba Abbasi, Hossein Karimi

**Affiliations:** 1 Department of Environmental Health Engineering, Genetic and Environmental Adventures Research Center, Shahid, Yazd, Iran; 2 Student Research Committee, Shahid Sadoughi University of Medical Sciences, Yazd, Iran; 3 Environmental Science and Technology Research Center, Department of Environmental Health Engineering, School of Public Health, Shahid Sadoughi University of Medical Sciences, Yazd, Iran; 4 Department of Operating Room, Paramedical School, Shahid Sadoughi University of Medical Sciences, Yazd, Iran; 5 Environmental Health Engineering, Shiraz University of Medical Science, Shiraz, Iran; 6 Student Research Committee and Department of Environmental Health Engineering, School of Health, Isfahan University of Medical Sciences, Isfahan, Iran

**Keywords:** Environmental sciences, Natural hazards, Diseases, Health care

## Abstract

This study was aimed to investigate the air pollutants impact on heart patient's hospital admission rates in Yazd for the first time. Modeling was done by time series, multivariate linear regression, and artificial neural network (ANN). During 5 years, the mean concentrations of PM_10_, SO_2_, O_3_, NO_2_, and CO were 98.48 μg m^−3^, 8.57 ppm, 19.66 ppm, 18.14 ppm, and 4.07 ppm, respectively. The total number of cardiovascular disease (CD) patients was 12,491, of which 57% and 43% were related to men and women, respectively. The maximum correlation of air pollutants was observed between CO and PM_10_ (R = 0.62). The presence of SO_2_ and NO_2_ can be dependent on meteorological parameters (R = 0.48). Despite there was a positive correlation between age and CD (p = 0.001), the highest correlation was detected between SO_2_ and CD (R = 0.4). The annual variation trend of SO_2_, NO_2_, and CO concentrations was more similar to the variations trend in meteorological parameters. Moreover, the temperature had also been an effective factor in the O_3_ variation rate at lag = 0. On the other hand, SO_2_ has been the most effective contaminant in CD patient admissions in hospitals (R = 0.45). In the monthly database classification, SO_2_ and NO_2_ were the most prominent factors in the CD (R = 0.5). The multivariate linear regression model also showed that CO and SO_2_ were significant contaminants in the number of hospital admissions (R = 0.46, p = 0.001) that both pollutants were a function of air temperature (p = 0.002). In the ANN nonlinear model, the 14, 12, 10, and 13 neurons in the hidden layer were formed the best structure for PM, NO_2_, O_3_, and SO_2_, respectively. Thus, the R_all_ rate for these structures was 0.78–0.83. In these structures, according to the autocorrelation of error in lag = 0, the series are stationary, which makes it possible to predict using this model. According to the results, the artificial neural network had a good ability to predict the relationship between the effect of air pollutants on the CD in a 5 years' time series.

## Introduction

Air pollution is an impressive parameter in various diseases including respiratory (RD) and cardiovascular disease (CD)^1,2^. Short-term and long-term exposure to these pollutants has led to signs of illness, recurrence or mortality, loss of life expectancy, and increased hospitalization of patients. Past studies have extensively addressed this issue^3–6,8^. Many models have been proposed to determine the relationship between air pollutants and the number of CD and RD patients admission^7,8^. Due to the increasing importance of variables affecting health and lack of suitable structural models for forecasting, on the other hand, Time series modeling has been developed. Time series contain a set of chronologically arranged evidence that can be predicted by arranging time-dependent observations^9^. Many of these studies have investigated the correlation, linear, and nonlinear relationships between these variables^10,11^. In the Slama (2019) study, the hospitalization rate of RD patients was estimated using correlation analysis and distributed lag non-linear model^12^. However, in Iran and many developing countries, less attention has been paid to this issue. Among the time series prediction methods, Artificial Neural Network (ANN) is more accurate because it is self-adaptive and data-based^13^. Also, ANN follows nonlinear mathematical relationships and is easily generalizable and used for various functions^14^. In the study by Rahman et al., the ANN's superiority over other linear and numerical methods for predicting hospital admissions due to air pollution has also been mentioned^15^. As well, Zhou et al., studied the ANN's superiority over other linear and numerical methods for predicting hospital admissions due to air pollution has also been mentioned. However, in many cities of Iran, including Yazd, a less predictive relationship between the effects of air pollutants on CD and RD patients has been investigated using ANN. Due to the importance of epidemiological studies on the effects of air pollution on human health and the location of Yazd city in desert areas of Iran with high dust flow, such studies are necessary. The ability of the ANN to determine nonlinear relationships between variables affecting the health consequences of air pollution can also be used in this city. Thus this study was aimed to evaluate the hospital admission rate because of CD by ANN and other statistical analyses and models in Yazd-Central Iran.

## Materials and methods

### Study area

**Figure 1:**
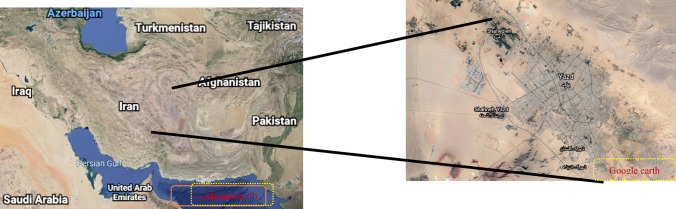
The geographical location of the study area. Maps of Iran-Yazd are available at: https://earth.google.com/web/search/iran/@32.48376657,54.07653009,1720.21296492a,2586706.88328266d,35y,-0h,0t,0r/data=CigiJgokCV-5_6ohZkBAEaVzQIn7uz5AGYK1zaKG3kxAIXKfRyghJEpA and https://earth.google.com/web/search/yazd/@31.87955845,54.33676713,1228.81847223a,31608.9548231d,35y,0h,0t,0r/data=Cm8aRRI_CiUweDNmYTYxOTkzMDM1YjJhOTE6MHg1ZDkyYTE5ZGQ3ZDRhMTBjGfKr3oa95T9AIfovxnatLUtAKgR5YXpkGAIgASImCiQJotZsLUxlQ0AR11C4YGjzNUAZMIgmcwbTU0AhwkoZJxXbPEA in Google earth (2020), respectively.

Yazd city is located in central Iran and geographical coordinates of 31.8974°N, 54.3569°E. Its population is 656,474 (2016), of which 267.73 are under the age of 14 years, 564,107 are between the ages of 14 and 65, and about 65,594 are over 65 years. It has an area of 76,469 m^2^, 1230 m above sea level, and has a warm and dry climate with extended deserts. The geographical location of the study area is obtained from Google earth and shown in Fig. 1.

### Data collection

The data was gathered from the hospital database, which is including the number, gender, and age of the patients, also the cause of the disease. These data were obtained from two large hospitals of Yazd, which are referral centers for heart patients. Also, air pollutant concentration data were obtained from the air pollutants monitoring station based in Yazd. Studied air pollution were included PM_10_, SO_2_, O_3_, NO_2_, and CO. Information was related to 5 consecutive years, from 2016 till 2019, and including 1559 data.

### The modeling of the relationship between pollutant and CD

In this study, the relationship between air pollutants and CD was analyzed by several models included linear model and non-linear. The linear model had included a linear regression model, multivariate linear regression model, and correlation. Besides, the time series of data performed by non-linear models such as ANN. Moreover, the relationship significant between these variables was predicted by the AMOS method.

#### The linear modeling

In this study, the relationship between dependent variables such as CD and independent variables included SO_2_, CO_2_, PM_10_, PM_2.5_, O_3_, and NO_2_ was identified by the regression model. So, the pollutant variation trend and the number of hospital admissions relative to the 5-year studied period were determined by the linear regression model. In this model, the relationship value between variables was determined by R-square.

Besides, the multivariate regression was used to generate a measure of effect, typically CD risks ratio due to air pollutants. So that it describes the associations between these variables. Moreover, the synergistic effect of pollutants on the number of admitted patients was determined using a multivariate regression model.

#### ANN modeling

In the current study, time series, relationships between variables, and outcomes analysis were done using ANN. The used algorithm in this study was Levenberg–Marqut. Furthermore, the nonlinear auto-regressive exogenous model (NARX) series available in ANN was used because of its higher accuracy than other series. The independent variable (air pollutants) and the dependent variable (CD admission) were entered into the model, as input and output, respectively. The hidden layers included intermediate variables formed by the ANN, which allows the modeling of complex relationships between variables. The transfer function in the hidden layer and the output layer was the sigmoid transfer function and linear transfer function, respectively. In this method, the modeling of a pollutant on the number of hospital admissions in each of the hospitals was investigated. Therefore, 70% of data is used for training, 15% for validation, and 15% for testing. Based on experience, the number of neurons and delays were selected in the range of 10 to 18 and 4 to 10, respectively. In total, five models were run for each pollutant. Ultimately, considering the 5 numbers of studied pollutants and the number of CD admissions in the hospital, 315 times mode were done. Finally, based on the mean square error (MSE), error rate, and correlation coefficient, the best structure in ANN for each pollutant was determined.

### Statistical analysis

In the current study, descriptive and analytical statistics were used for data analysis. Concentrations of pollutants and the number of patients were shown using mean and dispersion rates. Data differences in each year were determined by *t* test. The correlation between the number of patients and other factors was determined by the Pearson correlation.

### Ethical approval and consent to participate

In the present study, the data recorded in the hospital archive were used. In order to access the essential data, the necessary permission was issued by the Shahid Sadoughi University of Medical Sciences and correspondence was made. Given that the data was without the names of individuals, there was no need for informed consent.

This study was conducted with the approval of Shahid Sadoughi University of Medical Sciences and Health Services, Medical Ethics Committee. Code: IR.SSU.SPH.REC.1397.164. This study was approved by Shahid Sadoughi University of Medical Sciences in Yazd, which was stated that there is no need to obtain informed consent from patients. The project was found to be in accordance to the ethical principles and national norms and standards for conducting Medical research in Iran. Finally, all authors confirm that all methods were performed in accordance with the relevant guidelines and regulations to human data.

## Results and discussion

**Figure 2:**
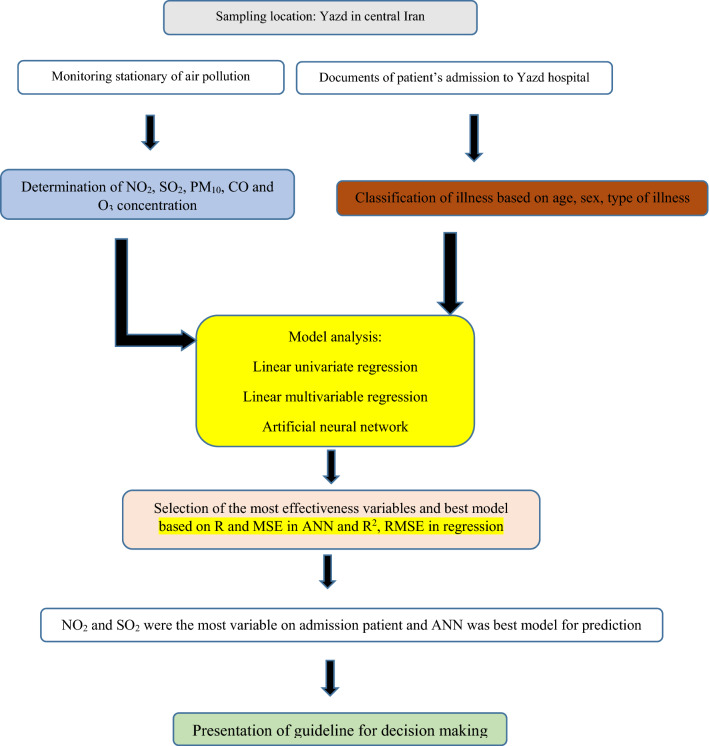
Methodological framework of study.

In this study, the effect of air pollutants in Yazd city on hospital admission rate because of CD disorder was investigated by using time series modeling. First, the current situation in this city was expressed, and then the relationship between each of the two variables was investigated. Ultimately, time series were predicted using ANN. The overall methodology of the study was shown in Fig. 2.

### The profiles of air pollutants and admission CD patients over 5 studied years

The profile of air pollutants and meteorological parameters over the 5 years were shown in Table 1.

**Table 1:** Mean, maximum and minimum concentrations of air pollutants over 5 years.

Air pollutants	Mean ± SD	Min	25th percentile	50th percentile	75th percentile	Max
PM_10_ (μg m^−3^)	98.48 ± 50.8	0.08	67.516	88.2135	117.978	3002
SO_2_ (ppm)	8.57 ± 4.3	0.4	5.41	7.7575	11.027	28.73
O_3_ (ppm)	19.66 ± 10.7	6.16	14.3	18.4	23.26	1224
NO_2_ (ppm)	18.1414	0.1	9.215	12.8815	21.21	91.94
CO (ppm)	4.07 ± 1.8	0.8	3.11	3.92	4.98	139.6
**Meteorological variables**						
Temperature (°C)	20.7313 ± 9.4	− 3.2	12.2	14.1	29.6	37.4
RH (%)	24.88 ± 16.67	4.375	12	20.125	32.625	96.625

According to Table 1, the mean concentration of PM_10_ was 98.48 ± 50.8 μg m^−3^, but the maximum was 3002 μg m^−3^. The results showed that during the 5 years of study, 1% of the samples were more than 500 µg m^−3^, while 63% of the data were more than the standard (100 µg m^−3^). Also, concentrations of SO_2_, O_3_, NO_2_, and CO were 28.73–0.4, 1224.6.16, 91.94–0.1, and 139.6–0.8 µg m^−3^, respectively. Additionally, 25% of the data for PM_10_ and O_3_ concentrations were in the range of 118–3002 and 23–1224 µg m^−3^, respectively. Because on some days of the year, the concentration of PM_10_ rises due to dust storms.

Moreover, the correlation coefficient between the concentrations of pollutants over the 5 years was shown in Table S1. The NO_2_ concentration correlation with meteorological factors was more than other pollutants. The correlation coefficient between air pollutants and meteorological parameters also showed that the linear correlation between them for PM_10_, NO_2_, O_3_, CO, and SO_2_ was (R_temp_ = − 0.16; R_humidity_ = − 0.2), (R_temp_ = 1; R_humidity_ = 0.42), (R_temp_ = − 0.19; R_humidity_ = − 0.3), (R_temp_ = 0.1; R_humidity_ = − 0.22), and (R_temp_ = 0.48; R_humidity_ = 0.39), respectively. In another study, there was a negligible and negative correlation between air pollutants and meteorological parameters^16,17^.

Among the index pollutants in Table S1, the highest positive and negative linear correlation was between CO and PM_10_ (R = 0.62) and CO and SO_2_ (R = − 0.65), respectively. In China, the highest correlation was detected between NO_2_ and PM_10_. Whereas, the lowest and negative relationships were observed between O_3_ and others^16^. The number of CD patients admitted pattern to Yazd hospital by sex and two age groups less than 65 years and above, was shown in Table 2.

**Table 2:** The mean, minimum and maximum number of CD patients by sex and age group.

	Total disease	Mean ± SD	Min	Max
**CDs**				
Total	2491	1.56 ± 2.4	1	24
Men	6598	4.99 ± 3.6	1	19
Women	5749	4.35 ± 3.24	1	18
0–64	2109	2.6 ± 2.9	1	24
More than 65	382	1.87 ± 1.7	1	14

Based on Table 2, the total number of CD patients over 5 years was 12,341. Of these, 57% were male (6598) and 43% were female (5749) (p < 0.0001). The mean total of CD patients over 5 years was 9.1, with a maximum CD of 27 people per day. On the other hand, 2109 CD patients were in the age group less than 65 years and 382 (15% of total CD) were more than 65 years of age, while the population over 65 years old constitutes 16% of the total population. There was a relatively low correlation between the number of CDs admitted and their age (R = 0.46). The relationship of univariate regression between pollutant concentration and CD count showed that this correlation was very low over 5 years. Thus, univariate linear regression models have not been a suitable model for predicting the time series of the effect of air pollutants on the number of hospital admissions of CD. The association of univariate regression between pollutant concentration and the number of CD patients showed that this correlation was very low over 5 years. So, for PM_10_, NO_2_, CO, SO_2,_ and O_3_ were (R = − 0.0444), (R = 0.0749), (R = − 0.0736), (R = 0.1617), and (R = − 0.052), respectively. The correlation between the concentration of pollutants and the number of CD admission during the 5 years in Yazd was shown in Table 3.

**Table 3:** The relationship between index pollutants and hospital admission rates of CD in different lags.

R	PM_10_	SO_2_	NO_2_	O_3_	CO
Lag = 0	− 0.21	0.4	0.27	− 0.23	− 0.27
Lag = 1	− 0.212	0.39	0.28	− 0.196	− 0.22
Lag = 2	− 0.18	0.37	0.269	− 0.217	− 0.268
Lag = 3	− 0.208	0.41	0.245	− 0.253	− 0.248
Lag = 4	− 0.226	0.398	0.235	− 0.2	− 0.17

According to Table 3, a univariate linear regression model showed that increasing SO_2_ and NO_2_ concentrations were effective in increasing hospital admissions. Hence, by increasing the lag, correlation with NO_2_ and SO_2_ decreased (lag1 = 0.28) and increased (lag 3 = 0.41), respectively. This correlation was reported in China for Ischemic stock^18^. The study of Ghozikali in Tabriz, northwestern Iran, and the study of Rajagopalan in JACC state also had higher SO_2_ and NO pollutants on hospital CD admission^19,20^. Moreover, in Arak-Iran in 2017, the highest effect was observed with NO_2_ at lag = 0. Besides, the highest effect of air pollutants on CD was related to CO in the single-pollutant model^16^. However, the low relationship can be due to the limitation of the linear regression model in considering the synergistic effects of the pollutants.

### Annual concentration of air pollutants and their correlation with CD disease admission

The annual distribution of air pollutant concentrations and the pattern of hospital admission for CD patients were shown in Table S2.

According to Table S2, the trend of O_3_ and PM_10_ variation were similar. The maximum mean concentrations of both pollutants in 2016 were 117.45 ± 69.7 μg m^−3^ and 22.8 ± 14.7 ppb, respectively. After this period, the concentration of the pollutants dramatically had increased. However, the maximum concentrations of PM_10_ and O_3_ were 3002 and 1224 ppb, respectively. On the other hand, the maximum standard deviation for both was 88.3 and 14.7 μg m^−3^, respectively. In the study of Ghorbani et al. in 2019 in Mashhad, with increasing concentrations of air pollutants, the mortality rate due to cardiovascular disease has increased. Remarkably, the highest relative risk was related to CO and SO_2_^21^. Also, in the study of Khajavi et al. In 2019 in Tehran using the AQI model, the highest effect of air pollutants on mortality due to cardiovascular disease occurred at lag = 0^22^.

The correlation between concentrations of air pollutants and meteorological parameters was shown in Table S3. The trend of detected changes for air pollutants can be due to fluctuations in weather. Because of the maximum mean O_3_ concentration and air temperature (37.4 °C) per year (Table S3), O_3_ concentration had a positive correlation with air temperature (R = 0.35–0.88). Because of the high temperature in this city, especially in the warm seasons (48–52 °C), the rate of photolysis of the NOx cycle was high, which produces more O_3_ in warm air. Additionally, the dissolution of O_3_ in water droplets in the air can also confirm the negative correlation of O_3_ with air humidity (R = − 0.33 to − 0.56)^23^. This trend has not been observed for other pollutants including CO, SO_2_, and NO_2_. Which the relationship between these pollutants and the meteorological parameters was not specific (Table 5). In general, climate conditions including temperature, and humidity did not change significantly (p > 0.05) during 5 years in Yazd. While concentrations of pollutants such as SO_2_, O_3_, NO_2_, and CO fluctuated during this period. Thus calculations were performed with lag = 1, 2, and 3 (Table 4).

**Table 4:** Correlation between CD hospital admission and annual concentration of indicator pollutants.

	Admission patients (R)				
	2015	2016	2017	2018	2019
**Lag = 0**					
PM_10_	0.377	− 0.266	− 0.197	0.233	− 0.237
NO_2_	− 0.839	− 0.289	0.207	0.130	0.669
O_3_	0.271	0.166	− 0.142	− 0.277	− 0.756
SO_2_	− 0.349	0.281	0.339	− 0.142	0.739
CO	− 0.730	− 0.225	− 0.248	− 0.098	− 0.614
**Lag = 1**					
PM_10_	0.322	− 0.274	0.057	0.158	− 0.253
NO_2_	− 0.814	− 0.072	0.238	0.204	0.668
O_3_	0.166	− 0.163	0.207	− 0.241	− 0.754
SO_2_	− 0.342	0.279	− 0.180	0.206	0.746
CO	− 0.686	0.085	0.160	0.090	− 0.609
**Lag = 2**					
PM_10_	0.113	− 0.212	0.297	− 0.263	− 0.276
NO_2_	0.296	− 0.099	0.294	− 0.338	0.657
O_3_	− 0.232	0.245	0.148	− 0.111	− 0.749
SO_2_	− 0.369	0.347	− 0.202	0.061	0.730
CO	0.254	− 0.212	0.200	0.339	− 0.607

The number of annual admissions of CD patients was variable during the study period (Table 4). In the concentration pattern of the pollutants, the maximum concentrations of SO_2_, O_3_, PM_10_, NO_2_, and CO were in months 3, 23, 7, and 3, respectively. The correlation between the number of CD diseases and the concentration of pollutants in these months was (R = 0.42; p = 0.0033), (R = − 0.20; p = 0.026), (R = − 0.026; p = 0.0161), (R = − 0.5; p < 0.0001), and (R = 0.37; p < 0.0001), respectively.

So, in 2015, 2016, 2017, 2018, and 2019 were 220, 353, 431, 421, and 1066, respectively. It can be said that this upward trend has grown so, in 2018 the number of these patients has increased to 3 times in 2017. However, there was no linear relationship between the annual concentration of pollutants and the number of CD hospital admissions at lag = 0, 1, and 2. There was also a low correlation for all pollutants (R^2^_SO2_ < 0.2). In addition, according to Table 4, no specific trends were observed for the annual concentration of the pollutants studied. In the study of Sharifi et al. in Tehran, the mortality rate due to cardiovascular disease had a significant relationship with the daily concentration of O_3_ and suspended particles^24^. The study of Khanjani et al. in 2019 in Tehran also showed that the most distinguished effect of air pollutants on mortality due to cardiovascular disease was related to NO_2_ and PM_10_ and occurred at lag = 0^25^.

### The pattern of monthly concentration of pollutants and the number of CD patient hospital admission

**Figure 3:**
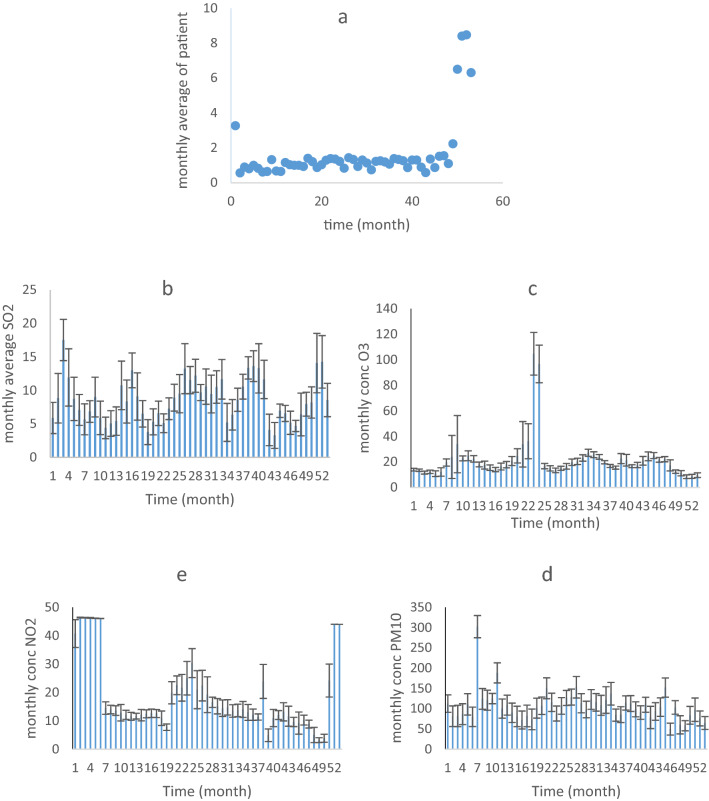
The monthly trend of standard pollutant concentrations and the number of CD hospital admissions.

The trend of monthly changes in pollutant concentrations and the number of hospital admissions per month for CD patients was shown in Fig. 3.

According to Fig. 3a, the mean monthly CD hospital admission number has changed slightly by the 50th month of the study. But immediately after this time, the number of admissions significantly increased (p < 0.05). Monthly concentrations of NO_2_ had the highest effect on the number of CD hospital admissions in Yazd. Meanwhile, the upward pattern in the number of CD patients was more consistent with the increase in NO_2_ concentration over the 50th week. This correlation was not observed for the early months of the study. The correlation between the number of CD patients and the monthly concentration of pollutants was given in Table 5.

**Table 5:** Correlation between concentration of indicator pollutants and the number of monthly CD patients admitted.

R	PM_10_	SO_2_	NO_2_	O_3_	CO
	− 0.48	0.51	0.50	− 0.44	− 0.49

By comparing Tables 4 and 5, it can be concluded that the monthly concentration trend has a better prediction for the relationship between the index pollutants and the admission of CD patients in Yazd. In the study of Liu et al., monthly results also showed higher accuracy in predicting the association between hospital admissions and air pollutants^26^. However, it can be concluded that linear models were not suitable for determining the relationship between air pollutants and CD hospital admissions. Consequently, the prediction was performed using multivariate linear regression models and ANN.

### Time series prediction of hospital admission numbers associated with air pollution by ANN and multivariate linear regression model

**Figure 4:**
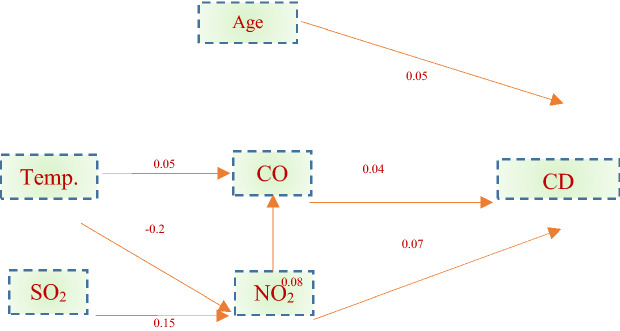
The relationship between index pollutants and the number of CD patients admitted in the hospital using multivariate linear regression.

In this study, the relationship between environmental pollutants and the number of CD patients admitted using the Stepwise regression model. A schematic of these relationships was shown in the regression model in Fig. 4.

In this model with Adjusted R^2^ = 0.21, F = 5.87, and p = 0.001, it was shown that the age of CD patients, CO and, NO_2_ had predictive power and were significant (p = 0.001)^19^. A study in Tabriz has also shown that most hospital admissions for CD patients, was related to age more than 65 years. Also, CO by forming COHb in the blood reduces the oxygen-carrying capacity of the blood, which was effective in causing CD diseases and heart failure^27–29^. In addition, NO_2_ has been identified as a pollutant representative of vehicle exhaust causing excitotoxicity, endothelial and inflammatory response, and damaging synaptic plasticity in the brain^30^.

Also, NO concentration and temperature on CO had explanatory power (p = 0.002) as the amount of CO produced from homes increases in winter. Temperature and SO_2_ had explanatory power over NO_2_ (p < 0.001), because at low temperatures, humidity increases, and as moisture increases, the NO_3_ in the air becomes HNO_3_, which falls like fine droplets. The temperature effect on NO_2_ also results from the NOx photolysis cycle. Due to the higher accuracy of nonlinear models such as ANN than linear regression models for air quality time series, determining the effect of index pollutants on numbers of CD patients admission in the hospital was done by ANN with the NARX model^31,32^.

The optimal models derived from the ANN used to predict the number of CD hospital admissions due to index pollutants were determined according to the NARX criteria and were shown in Table S4. According to Table S4, the best model to predict the effects of PM_10_, NO_2_, O_3_, and SO_2_ had 14, 12, 10, and 13 neurons in the hidden layer. In these structures, the delay has been 6, 5, 9, and 9, respectively. Correlation coefficients for PM_10_, NO_2_, O_3_ and SO_2_ were 0.78, 0.79, 0.81, and 0.83, respectively. As such, the ANN had high power in predicting the number of hospital admissions for SO_2_-induced CD disease^33^ because SO_2_ is a gaseous pollutant in the environment. Which increases the expression of the proinflammatory enzyme and vasoregulatory pathway and is effective in the development and progression of many CD diseases^34,35^. Whereas NO_2_, O_3_, and NO have been effective factors in increasing the admission of CD patients in Tabriz^36^. In addition, the error autocorrelation function plot of the various series in Fig. S1a showed that the optimal models used in this study were stationary due to the maximum correlation in lag = 0 and with a 95% confidence limit. Thus, this model can be used to predict hospital admissions due to air pollutants.

Besides, the time-series response plot in Fig. S1c also shows that the output curve was distributed on both sides of the response curve. The low error rate in training, testing, and validation showed that the ANN model was reliably reflected in time series data. Only in 2019, this error was more than the previous time. Other studies have also shown that ANN has been a good model for predicting the admission of CD patients to hospitals in Iranian cities and other countries, compared to logistic regression and NARX methods^19,30,37,38^. According to the results of regression modeling and ANN, SO_2_ has been an effective factor in hospital admission of CD patients in Yazd. Which has not received considerable attention in previous studies in the central region of Iran. In addition, in this study, the effect of single indicator pollutants by ANN was investigated but considering their synergistic effect, determine the effect of other specific pollutants and synergistic effects are suggested. According to the results of the current study, in this type of modeling, the correlation coefficient was less than 0.9. However, the results obtained from the used models had a higher relationship for the linear regression models (R = 0.45).

## Conclusion

In the current study, the effect of index pollutants in the air of Yazd-center of Iran on the number of hospital admissions of CD patients in a 5-year has been investigated, and the following results have been obtained:

According to the average concentration of pollutants, air quality in this period has not been in good condition. The changes trend in the concentration of NO_2_ and SO_2_ pollutants was more dependent on meteorological parameters. Also, most of the cardiovascular patients in this period were related to men. In this study, several models were examined to determine the relationship between air pollutants and the number of cardiovascular patients admitted to the hospital. According to the linear regression model, the highest correlation was in lag = 4 and for SO_2_, this correlation was lower than 0.5. Although the results of ANN have been appropriate, due to the limitations for the authors to predict these relationships using other algorithms, models and algorithms are proposed.

## Supplementary Information

Supplementary Information.
