# Supplementary material for: Ambient air pollution and cardiovascular disease rate an ANN modeling: Yazd-Central of Iran
Source: Sci Rep. 2021 Aug 20;11:16937. doi: 10.1038/s41598-021-94925-8 (PMC8379244; doi:10.1038/s41598-021-94925-8)
Supplement: Supplementary file 1 — Supplementary Information. [file 41598_2021_94925_MOESM1_ESM.docx]

##

b

a

PM10

SO2

##

d

c

O3

NO2

##

f

SO2

e

PM10

##

h

g

O3

NO2

## Fig. 1S. Error auto regression plot and time-series response for optimal model ANN.

Table S1. Correlation between air index pollutants and meteorological variables

| R | PM_10_ | NO_2_ | SO_2_ | O_3_ | CO | Temperature | Humidity |
| --- | --- | --- | --- | --- | --- | --- | --- |
| PM_10_ | 1.0 | -0.16 | -0.14 | 0.48 | 0.62 | -0.16 | -0.2 |
| NO_2_ | - | 1.0 | 0.48 | -0.19 | 0.1 | 0.97 | 0.42 |
| SO_2_ | - | - | 1.0 | -0.25 | -0.65 | 0.48 | 0.39 |
| O_3_ | - | - | - | 1.0 | 0.2 | -0.19 | -0.3 |
| CO | - | - | - | - | 1.0 | 0.1 | -0.22 |
| Temperature | - | - | - | - | - | 1.0 | 0.42 |
| Humidity | - | - | - | - | - | - | 1.0 |

Table S2. Mean, maximum and minimum concentrations of air pollutants and meteorological parameters for each year

| 2015 | | | 2016 | | | 2017 | | | 2018 | | | 2019 | | |  |
| --- | --- | --- | --- | --- | --- | --- | --- | --- | --- | --- | --- | --- | --- | --- | --- |
| Mean±SD | Min | Max | Mean±SD | Min | Max | Mean±SD | Min | Max | Mean±SD | Min | Max | Mean±SD | Min | Max |  |
| Air pollutant | | | | | | | | | | | | | | | |
| 92.17±47 | 25.2 | 357.5 | 103±88.3 | 29.6 | 3002 | 117.45±69.7 | 27.5 | 731.7 | 103.5±77.4 | 16.68 | 116.87 | 86.72±50.4 | 8 | 369.05 | PM_10_ |
| 10±5.57 | 0.62 | 28.5 | 7.47±3.97 | 0.49 | 28.7 | 8.59±3.86 | 0.42 | 20.9 | 9.85±4.1 | 1.69 | 28.7 | 7.73±4.2 | 1.17 | 24.5 | SO_2_ |
| 12.4±2.12 | 6.2 | 26.2 | 20.67±11.9 | 10.98 | 142.7 | 22.8±14.7 | 9.3 | 1224 | 20.98±4.35 | 13.5 | 34.7 | 17.03±6.69 | 6.2 | 29.67 | O_3_ |
| 43.2±11.9 | 0.01 | 91.94 | 12.58±4.29 | 8.5 | 46.08 | 19.68±9.7 | 5.79 | 87.6 | 13.11±6.5 | 2.4 | 49 | 14.5±14.2 | 0.98 | 44.06 | NO_2_ |
| 3.94±1 | 0.71 | 6.8 | 4.9±1.07 | 0.75 | 140 | 4.7±2.5 | 0.66 | 139.5 | 3.01±1.3 | 0.66 | 6.6 | 3.5±0.68 | 0.8 | 5.47 | CO |
| Meteorological parameters | | | | | | | | | | | | | | | |
| 21.5±6.3 | 2 | 29.5 | 21.4±9.5 | 2 | 37.3 | 21.1±9.8 | -3.2 | 37.4 | 21.56±9.1 | 0.6 | 36.2 | 22.6±9 | 2.1 | 37.1 | Temp. |
| 23.17±18 | 9.37 | 90.12 | 23.9±16.2 | 5 | 96.6 | 24.4±16.6 | 4.4 | 90.4 | 21.18±14.25 | 4.75 | 90.37 | 24.37±16.5 | 5.6 | 90.1 | RH |
| CD | | | | | | | | | | | | | | | |
| 1.23 | 0 | 14 | 0.97 | 0 | 5 | 1.18 | 0 | 6 | 1.15 | 0 | 6 | 3.34 | 0 | 24 |  |

Table S3. Correlation between concentrations of air pollutants and meteorological parameters

| R | 2014 | | 2015 | | 2016 | | 2017 | | 2018 | |
| --- | --- | --- | --- | --- | --- | --- | --- | --- | --- | --- |
|  | Temp. | Moist. | Temp. | Moist. | Temp. | Moist. | Temp. | Moist. | Temp. | Moist. |
| Lag = 0 | | | | | | | | | | |
| PM_10_ | 0.55 | -0.61 | 0.33 | -0.24 | -0.17 | 0.32 | 0.03 | -0.2 | 0.28 | 0.14 |
| NO_2_ | -0.75 | 0.56 | 0.17 | 0.1 | 0.58 | -0.52 | 0.36 | -0.24 | -0.75 | 0.35 |
| O_3_ | 0.35 | 0.071 | 0.57 | -0.47 | 0.45 | -0.33 | 0.66 | -0.47 | 0.84 | -0.56 |
| SO_2_ | -0.56 | 0.45 | -0.58 | 0.18 | 0.23 | 0.25 | -0.59 | 0.13 | -0.78 | 0.53 |
| CO | -0.69 | 0.3 | -0.15 | 0.19 | 0.3 | -0.44 | -0.59 | 0.35 | 0.80 | -0.54 |
| Lag = 1 | | | | | | | | | | |
| PM_10_ | 0.7 | - 0.56 | 0.11 | - 0.29 | - 0.16 | 0.33 | 0.29 | -0.25 | 0.32 | -0.23 |
| NO_2_ | -0.86 | 0.56 | 0.19 | -0.08 | 0.43 | -0.51 | 0.35 | -0.27 | -0.76 | 0.34 |
| O_3_ | 0.67 | -0.12 | 0.1 | -0.46 | 0.45 | -0.36 | 0.68 | -0.51 | 0.83 | -0.54 |
| SO2 | -0.76 | 0.33 | -0.59 | 0.44 | 0.21 | 0.37 | -0.59 | 0.04 | -0.78 | 0.55 |
| CO | -0.86 | 0.34 | 0.15 | -0.07 | 0.47 | -0.43 | -0.6 | 0.33 | 0.80 | -0.54 |

Table S4. Optimal models for predicting the number of hospitalized CD patients due to index pollutant

|  | Structure | Delay | Training MSE | Validation MSE | Test MSE | R_all_ | Autocorrelation of error in lag=0 | Epoch |
| --- | --- | --- | --- | --- | --- | --- | --- | --- |
| PM_10_ | 1:14:1 | 6 | 2.01 | 2.94 | 3.65 | 0.78 | 2.4 | 20 |
|  |  |  | R= 0.79 | R = 0.75 | R = 0.74 |  |  |  |
| NO_2_ | 1:12:1 | 5 | 2 | 3 | 2.75 | 0.79 | 2.3 | 6 |
|  |  |  | R = 0.82 | R = 0.8 | R = 0.65 |  |  |  |
| O3 | 1:10:1 | 9 | 1.75 | 2.48 | 3.02 | 0.81 | 2 |  |
|  |  |  | R = 0.85 | R = 0.62 | R = 0.71 |  |  |  |
| SO_2_ | 1:13:1 | 9 | 1.29 | 7.1 | 5.8 | 0.83 | 1.7 | 23 |
|  |  |  | R = 0.89 | R = 0.62 | R = 0.75 |  |  |  |
